# ATTITUDINAL DISTINCTIONS OF SOLO SEX ENGAGERS AMONG OLDER ADULTS

**DOI:** 10.1093/geroni/igae098.2863

**Published:** 2024-12-31

**Authors:** Janie Steckenrider

**Affiliations:** Loyola Marymount University, Los Angeles, California, United States

## Abstract

Solo sex gets little attention in the research on sexual activity of older adults as most studies frame definitions around partnered sexual behaviors. This does not reflect the reality of many older adults who lack active sexual partners due to death, dysfunction, or serious illness. When solo sex is included in studies, they rarely go beyond frequency. The aim of this study was to determine how sexually active older adults view solo sex, its importance, and attitude distinctions between solo sex engagers and non-engagers through survey research with a sample over age 60 (N=556). The majority (58%) of older adults engage in solo sex with men more likely (67%) than women (53%). As expected, solo sex engagers are twice as likely to use sexual toys and to be interested in using them if they currently do not. More solo sex engagers (70%) view orgasms as being extremely/very important for a good sexual experience than non-engagers (55%). Positive sexual outlooks correlate with solo sex as 80% of engagers anticipate an unlimited number of future orgasms compared to 65% of non-engagers. The most striking attitude distinction is 72% of engagers believe solo sex can be “great sex” compared to only 39% of non-engagers. Solo sex has psychological and physiological benefits for older adults and this study demonstrates solo sex correlates with positive sexual attitudes. The findings of this study highlight the need for further attention to the importance of solo sex for older adults and how it relates to positive sexual health.

